# Bulk versus Interface Nucleation of CO_2_ Hydrates from Computer Simulations

**DOI:** 10.1021/acs.jpcb.5c07607

**Published:** 2026-03-23

**Authors:** Joanna Grabowska, Samuel Blazquez, Carlos Vega, Eduardo Sanz

**Affiliations:** † Department of Physical Chemistry, Faculty of Chemistry, 431446 Gdansk University of Technology, ul. Narutowicza 11/12, Gdansk 80-233, Poland; ‡ Dpto. Química Física I, Fac. Ciencias Químicas, 16734 Universidad Complutense de Madrid, Madrid 28040, Spain

## Abstract

Gas hydrates are of great relevance to both the oil industry and the environment. Understanding how these solid structures nucleate from aqueous solutions is essential for controlling their formation. Experimental studies have often suggested that hydrate nucleation originates at the interface between the aqueous phase and the guest-molecule reservoir. To assess this hypothesis, we perform molecular dynamics simulations of CO_2_ hydrate nucleation. First, we place hydrate seeds at different positions relative to the interface and monitor their evolution, finding that seeds embedded in the bulk are more likely to grow than those located near or at the interface. Second, we analyze spontaneous nucleation simulations with and without an interface. Our previous work showed that nucleation rates are indistinguishable in both systems, strongly indicating that the interface does not play a role. Here, trajectory analysis reveals that hydrates nucleate in regions of locally high CO_2_ concentration, which arise spontaneously in the bulk and are not associated with the interface. Our results indicate that hydrate nucleation does not preferentially occur at the interface, at least under the deep supercooling conditions explored in this work. Further work at higher temperatures, and considering alternative nucleation locations, is needed to reconcile experiments and simulations, and thereby reach a deep understanding of the mechanism of hydrate formation.

## Graphical abstract

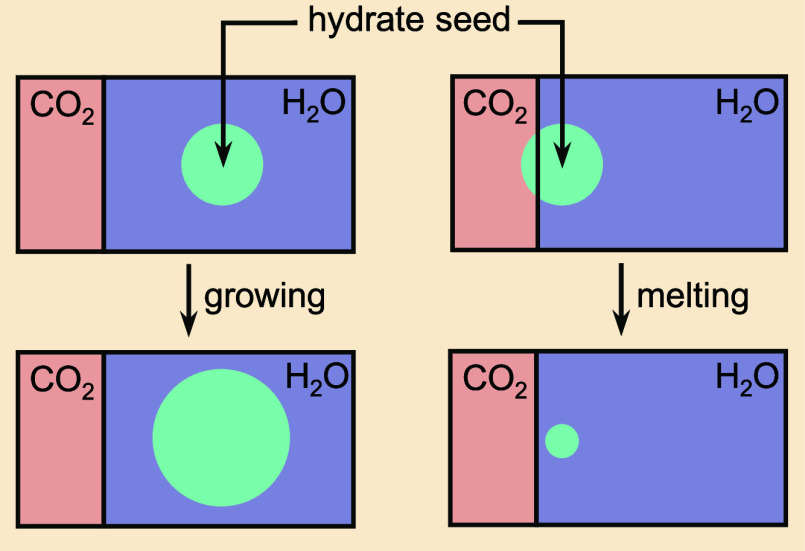

## 1 Introduction

Clathrate hydrates are crystalline structures in which gas molecules are trapped within cages of water molecules.^1^ Methane hydrates are primarily found in deep-sea sediments and permafrost regions, and serve as a substantial reservoir of methane and other fuel molecules. In addition to their potential as an energy source, gas hydrates offer a promising avenue for carbon dioxide (CO_2_) sequestration, as CO_2_ could replace methane within hydrate structures,^2–5^ providing a dual benefit of reducing greenhouse gas emissions while facilitating methane extraction. Hydrogen hydrates are also important because they offer a potential method for safe and compact storage of hydrogen, supporting the transition to cleaner energy sources. Methane hydrates, however, pose significant challenges for the oil and gas industry, particularly due to their tendency to form blockages in pipelines during hydrocarbon production and transportation, leading to operational inefficiencies and safety risks.^6^ It is thereby crucial to characterize and understand the formation and stability of hydrates.

Numerous experimental studies^7–15^ have been conducted to better understand the formation, growth and dissociation of gas hydrates under various thermodynamic and kinetic conditions. Researchers have investigated the effects of temperature, pressure, gas composition and the presence of inhibitors or promoters on hydrate crystallization and stability.

There seems to be compelling experimental evidence that hydrates nucleate at the interface between the solution and the hydrate former reservoir.^1,16–21^ This is a likely scenario given that the solubility of hydrate former molecules (CO_2_, CH_4_, H_2_S etc.) in water is typically much lower than the proportion of gas former molecules in the solid hydrate, which is 1 molecule per 5.75 water molecules in a perfect sI hydrate lattice. Thus, it appears reasonable that only in the vicinity of the interface there is enough hydrate former concentration to nucleate the hydrate. Experimental techniques, however, do not enable a direct visualization of the first hydrate embryo that nucleates from a disordered molecular arrangement, given its nanoscopic size and its short lifespan.

A theoretical approach is very useful for understanding and rationalizing the competition between homogeneous and interfacial nucleation; however, the values of the relevant parameters that enter the theoretical description require experimental validation, and the theory does not provide a molecular-level representation of the nucleation process.^22^ Molecular simulations can help bridge the gap in our understanding of nucleation at a molecular scale,^23–48^ particularly so with the emergence of realistic water models that have proven to be highly accurate in predicting the equilibrium behavior of real hydrates.^49–53^ Most simulation studies of hydrate nucleation, however, focus on the homogeneous nucleation of the hydrate in the bulk aqueous solution^27,30,31,34,38,39,54–56^ (in many cases with a guest molecule concentration way higher than the saturation concentration to enhance the nucleation rate) and the hypothetical location of the nucleus at the interface has not been properly assessed.

Some clues regarding the location of hydrate nucleation can be found in refs 57 and 58. In ref 57, focused on unveiling the molecular path leading to the formation of hydrates (blobs of guest molecules give rise to hydrate nuclei), it was mentioned that nuclei can indistinctly appear in the bulk or at the interface. According to ref 58, in contrast, nucleation in the bulk is not considered as a possibility (it either takes place at the interface with the hydrate-former rich phase or with a solid substrate at low and high temperatures respectively).

The fact that homogeneous nucleation cannot compete with nucleation at the interface is inconsistent with our recent simulation study.^55^ In ref 55 we conducted simulations of spontaneous CO_2_ hydrate nucleation from a bulk CO_2_ saturated solution and from a CO_2_ saturated solution in contact with a CO_2_ reservoir through a flat interface. The frequency of hydrate nucleation was the same in both cases, suggesting that the interface does not promote nucleation, at least under the studied conditions (245 and 250 K, 400 bar).

In this work, we use Molecular Dynamics (MD) simulations to clarify whether hydrates preferentially appear at the solution-hydrate former interface. More specifically, we place CO_2_ hydrate seeds at different locations relative to the CO_2_-solution interface and track their evolution in constant pressure, constant temperature (NpT) MD simulations. We find that, in the conditions of our study (400 bar and 255 K), proximity to the interface hinders the growth of CO_2_ hydrate seeds, leading to faster nucleation rates in the bulk than at the interface.

Our work highlights that hydrate nucleation in the bulk aqueous solution is faster than at the interface with the hydrate former. This result seems to be at odds with the general understanding that nucleation is faster at the interface.^1,16–21^ A study at higher temperatures (closer to the dissociation temperature, where experiments are typically carried out) is needed to explore the possibility of a crossover of nucleation location along temperature. Also, nucleation at the container walls or assisted by impurities are possibilities that could reconcile our results with the widely visualized observation of the appearance of hydrates at the interface in experimental research.

## 2 Methodology

### 2.1 Simulation Details

Water and CO_2_ molecules are modeled with TIP4P/Ice^59^ and TraPPE,^60^ respectively. Water–CO_2_ dispersive interactions are treated with the modified Lorentz–Berthelot rule proposed by Míguez et al. (which simply consists of multiplying by 1.13 the cross energy parameter given by Lorentz–Berthelot).^50^ These potentials yield accurate predictions of the CO_2_ hydrate three-phase line, with the dissociation temperature *T*_3_ at 400 bar matching the experimental value (286 K in experiment versus 290 K in simulations).^61^ The model, however, is not perfect. For instance, it overestimates the solubility of CO_2_ in liquid water at low temperatures.^62^ Importantly, while this force-field limitation affects the absolute value of the CO_2_ solubility, it does not alter the qualitative conclusions of the present work. All simulations were performed using the same force field, and comparisons between bulk and interfacial nucleation were therefore made under internally consistent conditions.

We focus on a pressure of 400 bar and most simulations are carried out at 255 K (i.e., 35 K supercooling). We focused on a pressure value typical of experiments in order to make predictions at experimentally relevant conditions and to enhance the solubility of CO_2_ in water. Other pressure values should be investigated to properly assess the role of this variable, that may affect the nucleation rate and pathway.^22^

MD simulations were used to obtain all the results presented in this work. The simulations were conducted with the use of the GROMACS package.^63,64^ The leapfrog algorithm with a time step of 2 fs has been used to integrate the equations of motion. Temperature was kept constant with the use of a Nosé–Hoover^65,66^ thermostat with a relaxation time of 2 ps, while pressure was kept constant with the Parrinello–Rahman^67^ barostat with the same relaxation time. The isothermal–isobaric ensemble (Np_*x*_T) has been used for most of the runs (with the barostat applied only along the *x* direction, which is normal to the interface); in some cases, NVT simulations were employed (details of the specific simulations will be provided below). For dispersive and Coulombic interactions, a cutoff of 1 nm has been used. Particle Mesh Ewald method^68^ has been used to compute electrostatic interactions. Long-range corrections for dispersive interactions were not used in our simulations.

All simulation systems were prepared using a two-phase system, where an aqueous solution of CO_2_ molecules was in contact - via a planar interface - with a CO_2_ reservoir. The size of this system was 11.6 × 8.5 × 8.5 nm^3^, where the *x* direction is perpendicular to the interface, and it contained 15735 molecules of water and 5 896 molecules of CO_2_.

The initial configuration of the two-phase system was prepared by combining pre-equilibrated simulation boxes of pure CO_2_ and of a CO_2_ aqueous solution (with CO_2_ molar fraction of 0.085, which is close to the expected equilibrium solubility of CO_2_ in water for the selected model under the studied conditions).^61^ The obtained system was then equilibrated for 40 ns at 255 K and 400 bar, in an anisotropic Np_*x*_T ensemble (i.e., the pressure was allowed to fluctuate only in the direction perpendicular to the interface). During the equilibration run, we monitored changes of the molar fraction of CO_2_ in the aqueous phase, which reached a stable value of approximately 0.077 after 20 ns. The equilibration run was continued for another 20 ns to ensure that the CO_2_ concentration in the aqueous phase remained at equilibrium.

After equilibration of the aqueous solution in contact with the CO_2_ reservoir, the Seeding method was used: CO_2_ hydrate crystal seeds of varying sizes were extracted from a bulk sI hydrate crystal in which all cages were fully occupied by CO_2_ molecules (the CO_2_ hydrate was equilibrated beforehand at 255 K and 400 bar for 10 ns) and inserted into the prepared two-phase system. After this step, the interface between the inserted cluster and the surrounding fluid was equilibrated with the use of three different protocols as explained in Supporting Information.

For all equilibration protocols, 6 types of systems were prepared, differing in the positioning of the seed with respect to the CO_2_-aqueous solution interface. The different seed locations are shown in the snapshots in Figure 1. Below, we indicate how we refer to each of these locations and briefly describe them:

1. *Bulk* (Figure 1a): the seed is inserted in the middle of the aqueous phase, which is the type of setup we used in our previous Seeding work.^55^
2. *Tangential* (Figure 1b): the seed is inserted tangentially touching the CO_2_-aqueous solution interface.
3. *3/4 in water* (Figure 1c): 1/4th of the seed diameter is immersed in the CO_2_ liquid and the remaining 3/4th is immersed in the aqueous solution.
4. *3/4 in water, cut* (Figure 1d): same as *3/4 in water* but the part of the seed immersed in the CO_2_ phase is cut off.
5. *1/2 in water* (Figure 1e): half of the seed is immersed in the CO_2_ phase and the other half in the aqueous solution.
6. *1/2 in water, cut* (Figure 1f): same as *1/2 in water* but the part of the seed immersed in the CO_2_ phase is cut off.

**Figure 1:**
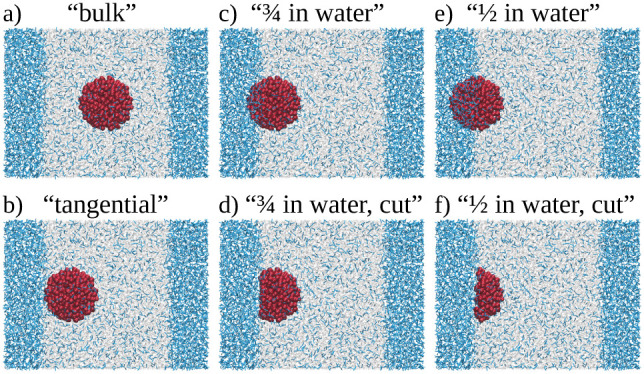
Snapshots of the seed locations and shapes investigated in this work: a) bulk, b) tangential, c) 3/4 in water, d) 3/4 in water, cut, e) 1/2 in water, f) 1/2 in water, cut. The color scheme of the figure is as follows: all molecules in the seed are represented as red spheres, water molecules and CO_2_ molecules which do not belong to the seed are represented as gray and cyan sticks, respectively. The seeds presented in the figure have a radius equal to 1.5 nm. The graphic was created using VMD^69^ software.

After the equilibration period we either fix the positions of atoms in the seed and let the seed grow or calculate the probability with which the unrestrained seed grows or shrinks. We determined the starting seed size as an average within the first 3 ns of a given production run.

To make sure that the *bulk* placement really corresponds to a molecular environment typical of a bulk aqueous solution we compute density profiles of CO_2_ and water across the interface. Such density profiles, shown in Figure 2, enable a microscopic identification of the interface based on the spatial variation of water and CO_2_ densities. As shown in the figure, deviations of the CO_2_ concentration in the aqueous phase from its bulk value are confined to a region extending approximately 0.7–0.8 nm from the CO_2_-rich phase. Beyond this distance, both the water density and the CO_2_ concentration reach plateau values characteristic of bulk aqueous solution. Based on this analysis, we defined the interfacial region as the zone in which the density and composition vary continuously between the CO_2_-rich and aqueous phases, and the bulk aqueous region as the region where these properties are spatially uniform. The hydrate seeds placed in the *bulk* configuration were positioned at distances greater than the extent of the influence of the interface on CO_2_ concentration (see the dashed circle in Figure 2). At these distances, the local environment is indistinguishable from bulk water in terms of density and CO_2_ concentration. Therefore, the hydrate seeds in systems labeled as *bulk* were not influenced by interfacial perturbations, while seeds placed closer to the CO_2_-rich phase clearly reside within the interfacial region.

**Figure 2:**
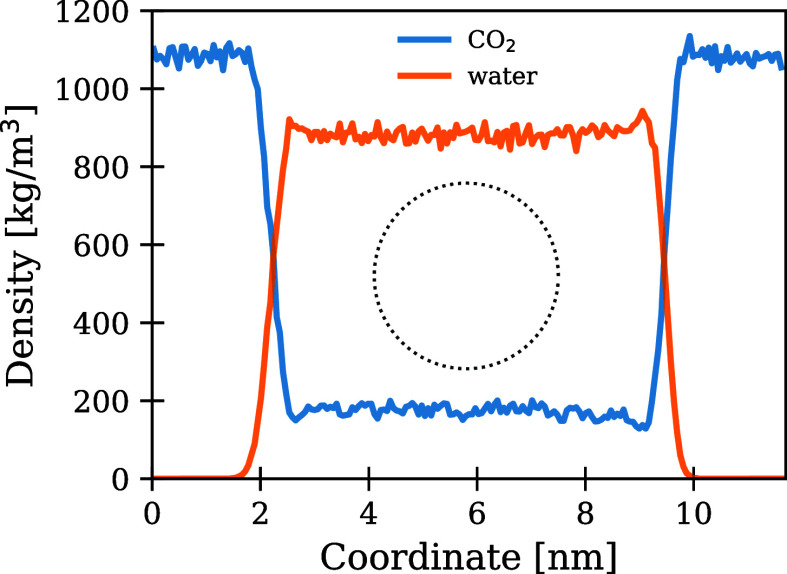
Density profiles of CO_2_ and water along the direction perpendicular to the CO_2_-water interface in the system used for seeding nucleation simulations. The circle marks the approximate spatial extent of the inserted hydrate seed of a radius of 1.7 nm and is included to provide a reference length scale relative to the profile gradients.

It is important to note that there is a substantial disparity between the CO_2_ concentration in the aqueous phase under the conditions studied (molar fraction ≈ 0.077) and the CO_2_ content in the hydrate phase (molar fraction ≈ 0.15). Under such conditions, the growth of hydrate-like structures could, in principle, lead to local CO_2_ depletion, potentially decelerating further growth unless rapid replenishment from a nearby CO_2_-rich phase occurs. In the present work, however, we focus on the nucleation regime and on the very early stages of critical nucleus growth. Within the time scales and cluster sizes probed by our seeding simulations, the CO_2_ concentration in the aqueous phase remains effectively constant. The inserted seeds do not grow large enough to induce measurable CO_2_ depletion in the surrounding solution, and consequently, mass transport limitations do not significantly influence the nucleation kinetics reported here.

## 3 Results

### 3.1 Simulations with Hydrate Seeds in Fixed Positions

As described in the Methods section, we used six types of systems in our study, which differed in the position and/or the shape of the inserted hydrate seed. To investigate the growth behavior of the inserted seeds we fixed the positions of all their constituent atoms during the simulations (performed at 255 K and 400 bar). As a result, seed growth was observed in all runs, regardless of their placement. Figure 3 shows the configurations from one of the runs for each type of seed placement, at 0, 20, and 40 ns. The main conclusion is that seeds with an initially spherical shape maintained an approximately spherical form, whereas seeds prepared as truncated spheres at the beginning of the run tended to acquire a spherical shape during the simulation. The tendency of the seeds to acquire a spherical shape suggests that the scenario of cap-shaped seeds nucleating on top of the CO_2_-aqueous solution interface^22^ does not accurately represent hydrate nucleation.

**Figure 3:**
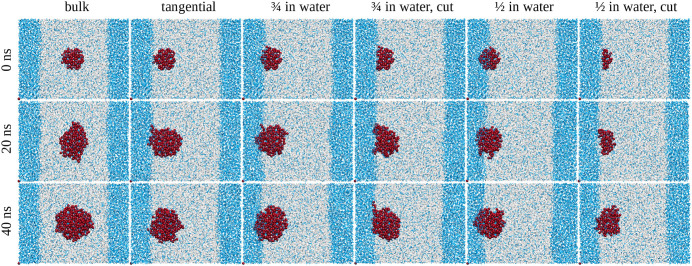
Configurations of the systems during the seeding simulations in which all molecules in the inserted hydrate seed were kept in fixed positions. Columns correspond to different types of seed locations whereas rows correspond to different times as indicated in the figure. Water molecules that were labeled as hydrate using a linear combination of *q̅*_3_ and *q̅*_12_ order parameter are presented as red spheres. Molecules of water in the liquid phase and molecules of CO_2_ are shown as gray and cyan lines, respectively. It can be observed that the seeds retain a roughly spherical shape during the simulations and those which were originally only a part of a sphere recover the spherical shape as the simulations progress. The growth of the seeds occurs mainly toward the liquid phase; in some cases, a shift of the position of the interface can be observed in order to accommodate the growth. Keep in mind that during these simulations no melting of the seeds is possible, since the molecules of the original seed inserted into the systems are kept in fixed positions during the simulations. The graphic was created using VMD^69^ software.

In Figure 4 the number of water molecules in the hydrate seed is plotted versus time for the different seed placements under study. In order to quantify the seed size we count the number of molecules of water it contains using a linear combination of *q̅*_3_ and *q̅*_12_ order parameters, as we also did in our previous work.^55^

**Figure 4:**
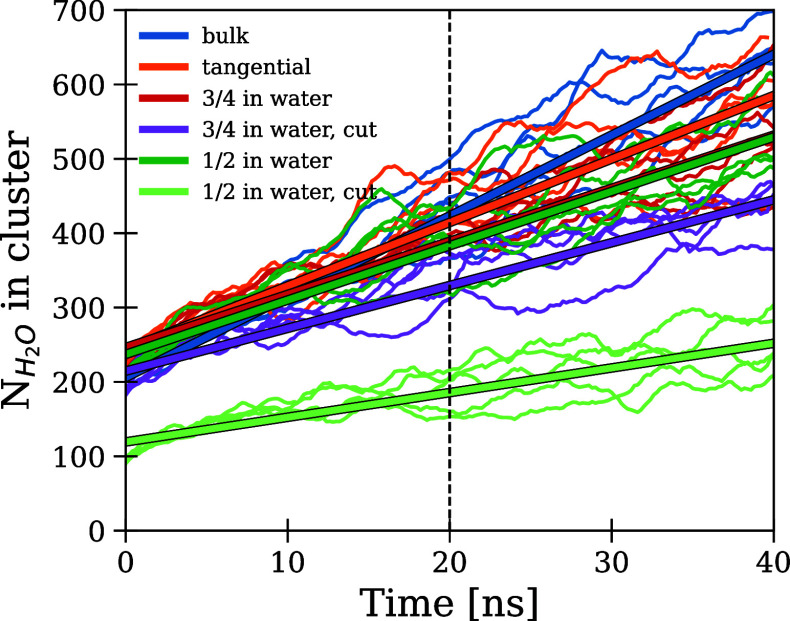
Changes of size of the hydrate seed during the seeding simulations in which all molecules in the inserted seed were kept in fixed positions. The radius of the seeds cut from the bulk hydrate was equal to 1.3 nm. Regression lines are included to show differences in the rates of growth of the seeds. The values of the slopes are (in 1/ns unit): 10.86 for *bulk*, 8.53 for *tangential*, 7.14 for *3/4 in water*, 5.75 for *3/4 in water, cut*, 7.30 for *1/2 in water* and 3.32 for *1/2 in water, cut*, respectively. The configurations obtained after 20 ns of simulation (indicated in the figure as a black dashed line) were used for the production runs (EQ3 equilibration protocol, described in Supporting Information).

The starting radius of all seeds was equal to 1.3 nm. Consequently, the starting size of the seed, measured as the number of water molecules it contains, is smaller in the systems where a portion of the sphere was cut off. The growth rate can be quantified through the slope of a linear fit to the $N_{H_{2}O}\left(t\right)$ curves. Such linear fits are shown with thick lines in Figure 4 and the specific values of the slopes are included in the caption of the figure. The growth was, on average, faster for the *bulk* and *tangential* seed placements. For the seed placements where the nucleus is partially immersed in the CO_2_-rich phase, *3/4 in water* and *1/2 in water*, the growth rate was a little slower, which indicates that the proximity of the interface decelerates the growth of the nucleus. The slowing down of the growth is even more pronounced for the systems where the seed was partially cut, although in these cases the comparison is not totally fair because the initial seed size is smaller. Since hydrates need water molecules to grow, it is expected that the growth is faster in the aqueous phase.

### 3.2 Simulations of the Unconstrained Hydrate Seeds

Under the same conditions as we used for the runs presented in the previous section—400 bar and 255 K—we carried out simulations in which the positions of the seeds inserted into the system were not constrained. This time seeds of different sizes were used, with a radius ranging from 1.3 to 2.0 nm. The starting configurations for these runs were equilibrated as described in Supporting Information, where we show that the specific protocol used for the equilibration of the seed interface does not affect the results.

An example of the evolution of the seeds after 40 ns is shown in Figure 5. As can be seen, the seeds originally inserted into the system close to, or immersed in, the CO_2_-rich phase tend to drift away from the interface into the aqueous solution. This observation clashes with the hypothesis that nucleation takes place at the interface.

**Figure 5:**
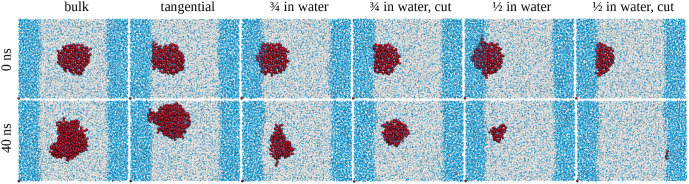
Snapshots of the system in unrestrained production runs at 0 and 40 ns. The starting radius of the seeds was 1.7 nm in all cases. The molecules of water that were found to belong to the biggest hydrate cluster (identified as in ref 55) are presented as red spheres. Water molecules in the liquid phase and CO_2_ molecules are shown as gray and cyan lines, respectively. The graphic was created using VMD^69^ software.

Depending on the location, shape and size of the seed at the beginning of the trajectory, the outcomes we observed were different. In some cases, the seeds were growing in most of the runs, in others they were almost always melting. To organize all these results, we analyzed changes in time of the seed sizes. The results are presented in Figure 6.

**Figure 6:**
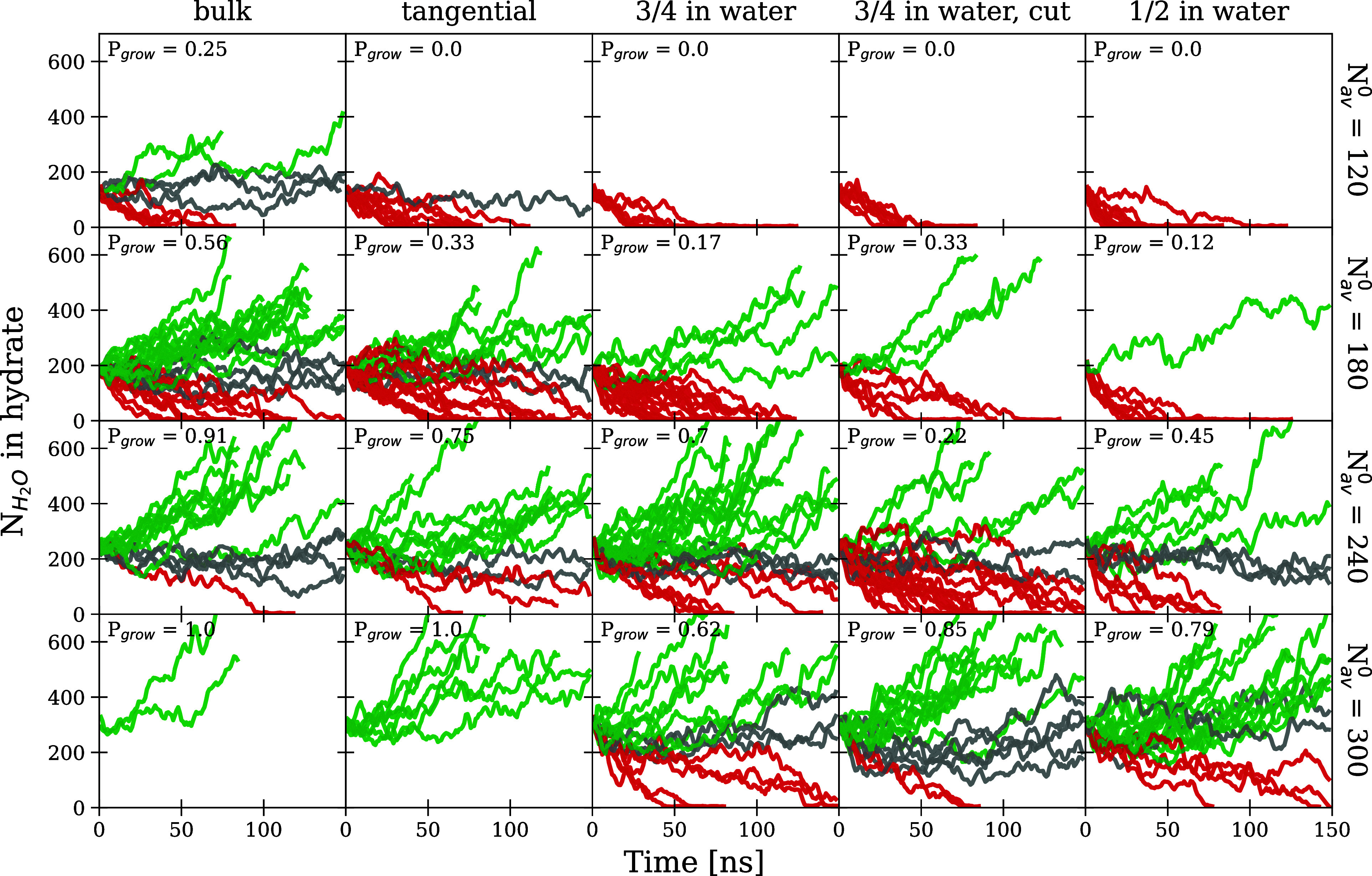
Changes in time of the size of the hydrate seed during production runs. Results are shown in separate rows according to the average size of the seed in the first 3 ns of a given production run. The average sizes of the seeds at the start of the runs presented within each row are indicated on the right-hand side of the figure. In the figure, the data for only a few starting seed sizes is shown (an extended figure with all initial sizes considered is presented in Supporting Information). The different seed locations/shapes are organized in different columns. Lines in the figure are colored in red if the seed melts and in green if it grows, while gray lines are used for the runs in which the size of the seed is stable (not changing enough to label it as either ″growing″ or ″melting″). The probability of growing was calculated in each plot based on the number of growing and melting seeds (the runs labeled as ″stable″ were not included in the calculations).

In the figure, the different seed locations are organized in columns, while rows correspond to a similar size of the seeds at the beginning of the trajectory (within a range of 20 water molecules around the average value shown on the right side of the figure). For clarity, only a few starting sizes of the seeds are included in Figure 6 (see Figure S2 in Supporting Information for the figure including all runs). The *1/2 in water, cut* type was not included in the figure, since in the majority of the runs, regardless of the starting seed size, the seeds were melting. Trajectories are colored in green and red if the seed size either increases or decreases significantly during the run (the change of the size must be greater than 50% compared to the starting size of the seed). Trajectories are colored in gray if the change of the seed size was smaller than 50%. The growth probability indicated inside each plot corresponds to the ratio between the number of green trajectories and the sum of green and red ones (gray ones are not considered for calculating the probability).

The growth probability increases as one moves down a given column. This trend is expected, since the size of the seed from which the trajectories are initiated increases down the column (the larger the seed is, the more likely it is that it grows).

By examining a particular row in Figure 6, one can clearly observe that the probability of growth decreases from left to right, as the seed is placed closer to the interface. The finding that the interface hinders the growth of hydrate seeds is one of the main results of this work.

As mentioned before, within each panel in Figure 6, we report the corresponding probability of growth. These probabilities are then plotted in Figure 7 as a function of the initial seed size for all seed locations and shapes considered. Although the curves are somewhat noisy due to limited statistics, one can clearly see that the cyan curve—corresponding to *bulk* spherical clusters—stands above the others, indicating that spherical clusters embedded in the bulk molecular environment have the highest tendency to grow. In contrast, the curves corresponding to the *1/2 in water* and *3/4 in water, cut* configurations (green and purple, respectively) fall below the rest, revealing that these locations are highly unfavorable. The *1/2 in water, cut* cluster type is even less favorable, but it does not appear in the figure because the growth probability was close to zero for all studied seed sizes. Overall, the results clearly demonstrate that clusters have a reduced chance of growing when placed near or at the interface.

**Figure 7:**
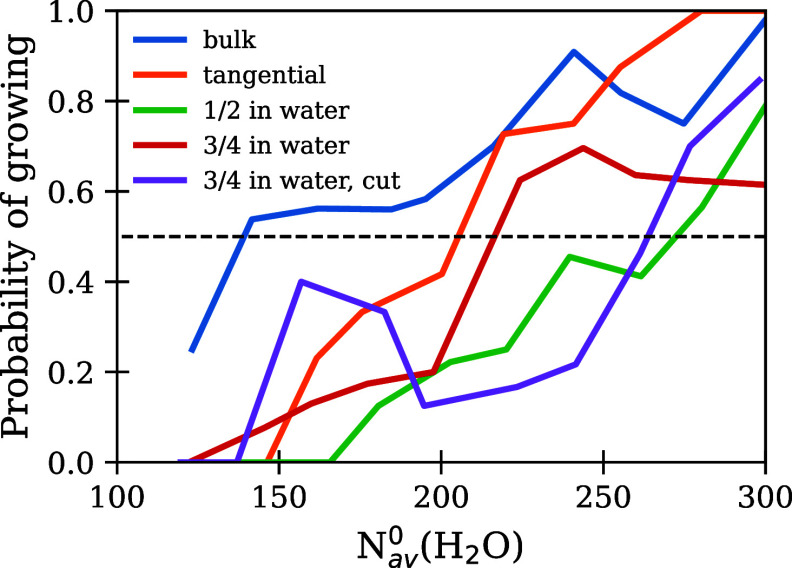
Probability of growing of hydrate seeds as a function of the initial seed size (measured as the number of water molecules belonging to the seed) for different system types (see legend).

Furthermore, truncating a cluster so that it presents a planar facet at the reservoir-solution interface does not promote growth. This becomes most evident when comparing the *1/2 in water* and *1/2 in water, cut* shapes: whereas clusters in the latter configuration (a hemisphere) never grew, spherical seeds reached a 50% growth probability for sizes larger than about 250 water molecules (see green curve in Figure 7). The observation that planar faces are disfavored is consistent with Figure 3, where we show that clusters spontaneously adopt a rounded shape as they grow. We emphasize that the comparison in Figure 7 is made at fixed numbers of water molecules in the seed. Had the comparison instead been carried out at fixed radius, truncated clusters would have had an even lower probability of growth, since they contain fewer molecules than their spherical counterparts (for a fixed radius).

### 3.3 Estimation of the Nucleation Rate for Different Seed Types

Based on Figure 7 it is possible to estimate the critical size of the seed types considered in this work. The horizontal dashed line in Figure 7 corresponds to a 50% probability. We identify the critical size, *N*_*c*_, as that for which the growth probability is 50% (the crossing between the horizontal dashed line and a given colored line). The smaller the critical cluster is, the higher the nucleation probability of a certain seed type. The estimated critical cluster sizes for most of the seed types examined in this study are reported in Table 1 (for the *1/2 in water, cut* configuration almost all clusters melted and no critical size could be determined, so we presume it is larger than the rest). From smallest to largest *N*_*c*_ the investigated seed types can be sorted as follows: *bulk*; *tangential*; *3/4 in water*; *3/4 in water, cut*; *1/2 in water*; *1/2 in water, cut*. This sequence confirms that bulk (homogeneous) nucleation is more favored than nucleation near or at the interface. Furthermore, it indicates that, at the conditions under study, spherical clusters are preferred to truncated ones (with the flat face lying on the reservoir-solution interface).

**Table 1:** Nucleation Rate of CO_2_ Hydrate in Water, *J*, Obtained from the Seeding Simulations at 255 K, 400 Bar, for Different System Types, and Parameters Used in Order to Obtain Nucleation Rates^a^ ^b^ ^c^ ^d^

	Bulk	Tangential	3/4 in water	3/4 in water, cut	1/2 in water
$N_{c}^{H_{2}O}$	140	205	217	264	272
$N_{c}^{{\mathrm{CO}}_{2}}$	24.3	35.7	37.7	45.9	47.3
Δ*G*_*c*_/(*k*_*B*_ *T*)	27.5	40.3	42.6	51.9	53.5
*J* (m^–3^ s^–1^)	1 × 10^23^	4 × 10^17^	4 × 10^16^	4 × 10^12^	8 × 10^11^

Parameters used for calculation of *J*	
*Z*	0.08
$f_{{\mathrm{CO}}_{2}}^{+}\left(s^{-1}\right)$	6.54 × 10^8^
$\rho_{L}^{{\mathrm{CO}}_{2}}\left(m^{-3}\right)$	2.6 × 10^27^

a The size of the critical cluster was estimated as number of water molecules belonging to the hydrate, with the use of linear combination of *q̅*_3_ and *q̅*_12_ order parameters.^55^

b The number of CO_2_ molecules in the critical cluster was then calculated by dividing $N_{c}^{H_{2}O}$ by a factor of 5.75, which corresponds to the water to CO_2_ ratio in a crystalline sI hydrate.

c Note that $N_{c}^{{\mathrm{CO}}_{2}}$ is equivalent to the number of cages given that we cut the seeds from a thermalised, fully occupied CO_2_ hydrate lattice.

d The estimated uncertainty for the critical nucleus size is ±20 water molecules (estimated from the bin width used to classify initial seed sizes), leading to relative uncertainties of about 5–10% in the nucleation barrier and to nucleation rate uncertainties ofapproximately 4–5 orders of magnitude.

Knowing Δμ_*N*_, the chemical potential difference between the crystal and the solution (which we calculated in our previous work for the same conditions, 255 K and 400 bar,^61^ and is equal to –2.26 k_B_T), we can then calculate the free energy barrier for nucleation, Δ*G*_*c*_, using the Classical Nucleation Theory^70–73^ result: Δ*G*_*c*_ = |Δμ_*N*_|*N*_*c*_/2. It is then possible to go further and estimate the nucleation rate using

1 $$J=\rho_{L}^{{\mathrm{CO}}_{2}}Zf_{{\mathrm{CO}}_{2}}^{+}\exp\left(\frac{-N_{c}^{{\mathrm{CO}}_{2}}\left|\Delta \mu_{N}\right|}{2k_{B}T}\right)$$

where $\rho_{L}^{{\mathrm{CO}}_{2}}$ is the number density of CO_2_ in the liquid phase, $N_{c}^{{\mathrm{CO}}_{2}}$ is the number of molecules of CO_2_ in the critical cluster, *Z* is the Zeldovich factor and $f_{{\mathrm{CO}}_{2}}^{+}$ is the attachment rate.

In order to estimate nucleation rates in our systems, we used the same values of *Z* and $f_{{\mathrm{CO}}_{2}}^{+}$ as in our previous work.^55^ $\rho_{L}^{{\mathrm{CO}}_{2}}$ is simply the number density of CO_2_ in the aqueous phase. The parameters used for the calculation of the nucleation rate, as well as the nucleation rate itself, are reported in Table 1. Note that the value we obtain for nucleation of *bulk* seeds (*J* = 10^23^ m^–3^ s^–1^) is consistent within 2 orders of magnitude with the value we published in ref 55 of *J* = 10^25^ m^–3^ s^–1^ (4–5 orders of magnitude is a typical error bar of Seeding nucleation rate calculations).^74,75^

These estimates, although rough, illustrate that the nucleation rate decreases by many orders of magnitude from a nucleus emerged in the bulk molecular environment to other less favorable locations close to or immersed in the CO_2_-rich phase. Even the nucleation in the second-best seed type, *tangential*, is about 6 orders of magnitude slower than bulk nucleation. When a spherical nucleus is placed with its equatorial line at the interface (*1/2 in water* location) one obtains nucleation rates more than 10 orders of magnitude slower than in the bulk. We do not find, therefore, any evidence of a preferred nucleation at the interface in our seeding simulations.

Let us revisit, however, the spontaneous nucleation simulations we performed in our previous work^55^ at lower temperatures (245 and 250 K) to check whether this finding is affected by the artificial Seeding of the hydrates cluster in the system.

### 3.4 Unseeded Spontaneous Nucleation

In previous work,^55^ we demonstrated that at 245 and 250 K under the pressure of 400 bar, spontaneous hydrate nucleation occurs within hundreds of nanoseconds. Furthermore, we found that the nucleation time is identical in a CO_2_-saturated bulk aqueous solution and in a two-phase system, where a slab of the aqueous solution is saturated with CO_2_ by contact with a reservoir through a flat interface (note that the CO_2_ concentration in the aqueous phase is the same in one-phase and two phase-systems). This finding clearly shows that, at least at these two temperatures, the presence of the interface does not accelerate nucleation. Moreover, this result also remarks that bulk-like behavior can be found in the aqueous slab of the two-phase system, as illustrated by the density profiles shown in Figure 2.

To corroborate this result, we analyze in this work the location of hydrate nuclei that appear spontaneously at 250 K in the two-phase system. According to the Seeding analysis performed in this work and to the spontaneous nucleation rate calculations conducted in ref 55, it is expected that these nuclei do not appear at the interface. In Figure 8 we show the center of mass location of the nucleus along time using a certain color code depending on the nucleus size (see legend) and for a selected spontaneous nucleation trajectory at 250 K. The horizontal dashed lines indicate the average location of the interface between the CO_2_-rich and the water-rich phases. Clearly, the nucleus does not appear close to any of the two interfaces, corroborating our main result that nucleation does not take place at the interface. A similar behavior was observed in all nucleating trajectories. In ref 76, where spontaneous CH_4_ hydrate nucleation was studied in systems containing a CH_4_ bubble embedded in the aqueous solution, they observed the appearance of the hydrate nucleus away from the bubble-aqueous solution interface, in agreement with what we see here for planar interfaces.

**Figure 8:**
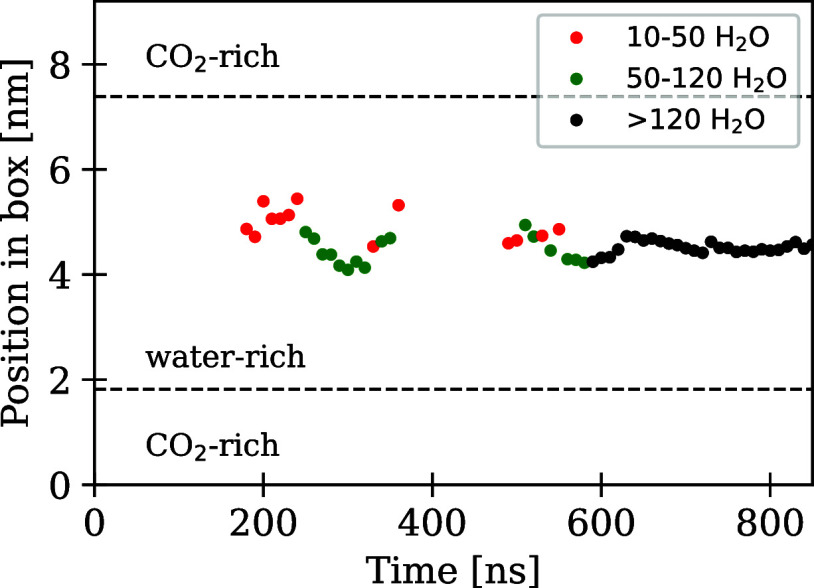
Dots indicate the coordinate perpendicular to the interface of the center of mass of the of the largest hydrate nucleus in a spontaneous nucleation trajectory at 250 K and 400 bar. The average location of the interfaces is indicated with the dashed horizontal lines. Different dot colors represent different size ranges of the nucleus (see legend), in terms of number of water molecules. Only sizes larger than 10 water molecules were considered.

In Figure 9 we show an analysis of the spontaneous nucleation path in bulk and two-phase systems. In the top part of the figure we show the number of CO_2_ molecules in the largest hydrate cluster identified with the MCG-3 algorithm.^77^ Below these plots, three snapshots of the systems at times specified by colored dots are presented.

**Figure 9:**
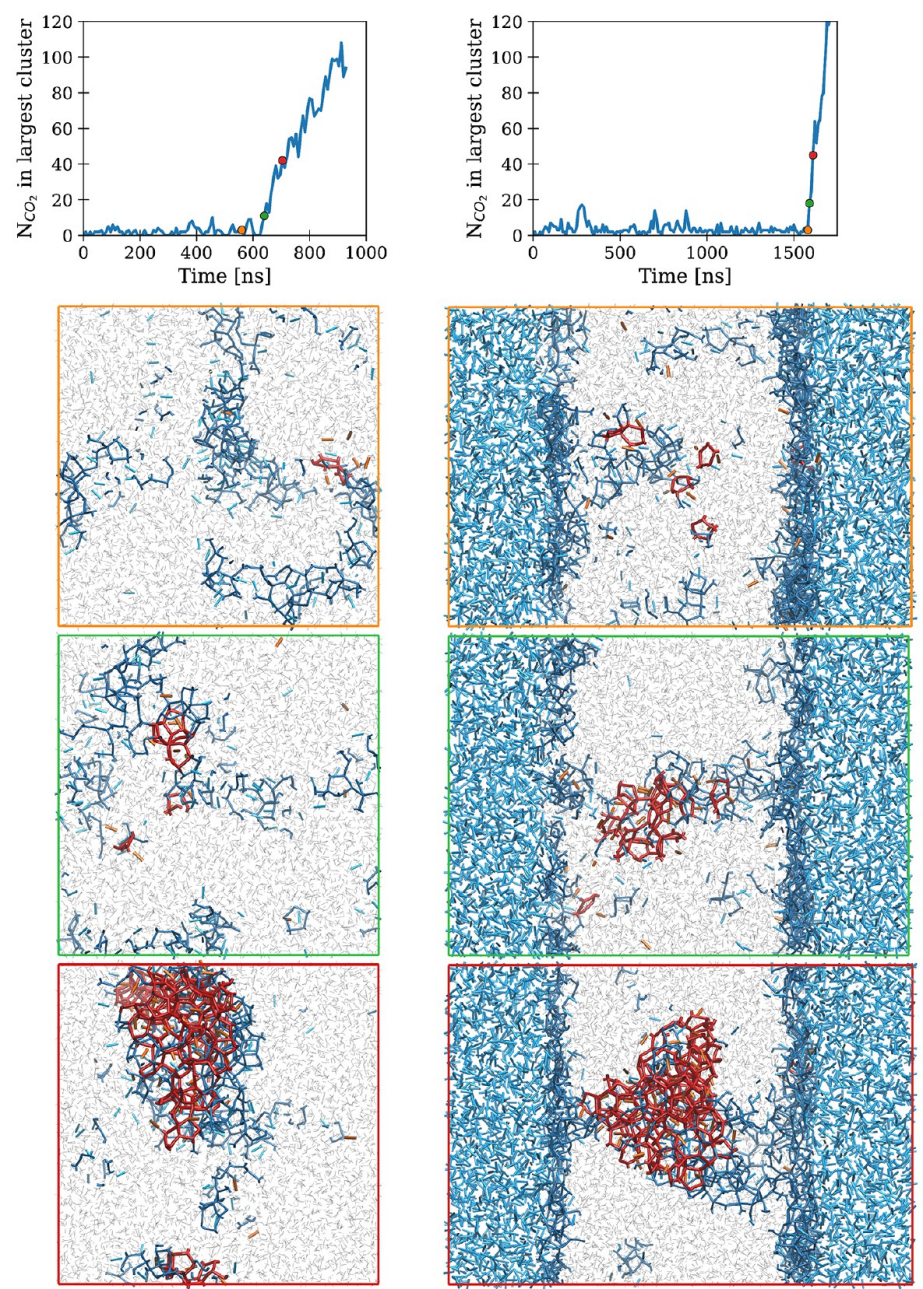
Top panel: size of the largest hydrate cluster in time in representative spontaneous nucleation trajectories in the one-phase and the two-phase systems at 400 bar and 250 K. For three moments in each trajectory, indicated by colored dots in the top plots, snapshots framed in the corresponding color are shown below. The clusters of hydrate recognized by the MCG-3 order parameter are marked with orange (CO_2_ molecules) and red (water molecules) bonds. Areas of locally higher density of CO_2_ are also shown, as light blue (CO_2_ molecules) and dark blue (water molecules) bonds. The areas of high CO_2_ density were found by looking for molecules of CO_2_ that have 11 or more neighboring molecules of CO_2_ (within distance of 0.9 nm). The graphic was created using VMD^69^ software.

For this particular analysis we chose the MCG-3 order parameter because it allows to identify the molecules of CO_2_ belonging to the hydrate seed, unlike the linear combination of *q̅*_3_ and *q̅*_12_ order parameters^55^ used in Table 1 which refers to water molecules. The MCG-3 order parameter labels a molecule of CO_2_ as hydrate if a set of geometrical criteria is met. First, a molecule of CO_2_ has to have a neighboring molecule of CO_2_ within 9 Å and this pair of CO_2_ molecules has to have at least 5 molecules of water ″shared″ between them. ″Shared″ water molecules, as described in ref 77, correspond to molecules of water inside an area limited by two cones located along the line connecting the two CO_2_ molecules and starting in the carbon atoms of CO_2_ molecules (semivertical angles of the cones are equal to 45°). Second, a molecule of CO_2_ can be labeled as hydrate only if it forms at least three pairs described in the previous step.

In the snapshots of Figure 9, molecules of CO_2_ labeled as hydrate by the MCG-3 order parameter and the molecules of water ″shared″ by pairs of CO_2_ molecules in the hydrate are highlighted in orange and red, respectively. Additionally, we searched for regions in the system where the concentration of CO_2_ was locally higher than the average. For that purpose, we found molecules of CO_2_ that have at least 11 neighboring CO_2_ molecules within 9 Å (the same distance criterion as for the MCG-3 order parameter) and highlighted them in cyan. For each pair of CO_2_ molecules found this way, we also highlighted (in dark blue) the molecules of water ″shared″ between them.

It is worth noting that MCG-3 does not label molecules of water as either hydrate or liquid. However, it enables identification of regions with a high local CO_2_ concentration and CO_2_ molecules in a hydrate-like arrangement. Thus, it is a suitable order parameter for our purpose of studying from a qualitative perspective the emergence of hydrates in regions of locally high CO_2_ concentration. For obtaining quantitative estimates of the nucleation rate (Section 3.3) we chose the linear combination of *q̅*_3_ and *q̅*_12_ proposed in ref 55, which was previously shown to provide seeding estimates of *J* in good agreement with spontaneous nucleation.^55^

As can be seen in the top part of Figure 9, in both system types - bulk and containing planar interface with a CO_2_ reservoir - there is an induction period of a few hundreds of nanoseconds where small clusters of hydrate form and redissolve until a stochastic fluctuation gives rise to a critical cluster that quickly grows.

In the snapshots we can see a similar nucleation mechanism in both cases. The presence of cyan-blue clustered regions before nucleation indicates that there is strong CO_2_ density heterogeneities. Obviously, in the two-phase system the interface corresponds to one of these regions, but there are also clear CO_2_ density fluctuations in the bulk aqueous phase. Interestingly, hydrate clusters always appear within a bulk high CO_2_ density regions. Thus, we envisage a mechanism where CO_2_ density fluctuations facilitate the emergence of hydrate clusters (in fact, spontaneous CH_4_ nucleation occurs when the solution is supersaturated with methane).^27^ Similar observation of nucleation in regions of high local guest molecule concentration has already been reported in previous works.^56,57,78^ However, our analysis reveals that these fluctuations are present both close to the CO_2_-aqueous solution interface and in the bulk aqueous phase, but the proximity of the interface disrupts the nucleation of the hydrate, making the bulk aqueous solution the preferred location for nucleation.

## 4 Discussion

Our simulations clearly show that nucleation preferentially occurs in the bulk. Does our work imply that hydrate nucleation also takes place in the bulk in real systems? Experiments observe hydrate appearance and growth at the interface.^16,18–21^ Here we provide some speculative hypotheses to reconcile our simulation results with experimental observations.

One possibility is that nucleation takes place in the bulk (homogeneous nucleation) and then the nucleus attaches to the interface, where it subsequently grows. However, this scenario seems unlikely because experiments generally observe hydrate nucleation at temperatures a few kelvin below the dissociation temperature,^13,14,18–20,79^ and several simulation studies estimate that homogeneous nucleation is not possible at such a small supercooling.^31,55^

Where, then, is nucleation actually taking place? One possibility is that there is a crossover from bulk (homogeneous) to interfacial (heterogeneous) nucleation as temperature increases. In our simulations, bulk nucleation of CO_2_ hydrates is faster at 245, 250, and 255 K. However, the homogeneous nucleation rate drops rapidly with increasing temperature. In contrast, the interfacial nucleation rate may have a weaker temperature dependence. This opens the possibility that at higher temperatures interfacial nucleation becomes dominant.

Another plausible scenario is that nucleation happens in the contact line between the aqueous solution, the hydrate-former rich phase and the cell walls. This possibility has been proposed in several papers^14,16,17,20^ and may also explain why higher supercoolings are typically observed when hydrate formation occurs in droplets suspended without contact with solid walls.^16,20,80^ Additionally, the presence of impurities, both in the bulk or trapped at the interface, may also increase the temperature at which hydrate formation is observed. Simulations show that solid surfaces can indeed promote hydrate nucleation.^58,81^

We emphasize that our work focuses on the early nucleation stage. Rapid growth may require a fast supply of hydrate-former molecules - CO_2_ in our case - from the hydrate-former-rich phase. Consequently, nucleation and early growth may occur in the bulk molecular environment (homogeneous nucleation), whereas sustained growth may require the postcritical nucleus to be in close proximity to the interface. The time and length scales required to investigate such growth processes are beyond those accessible to our molecular simulations.

Our work appears to clash with a recent simulation study where nucleation is reported to take place at the interface at high supercooling.^58^ A possible difference between our work and that of ref 58 is that they use H_2_S instead of CO_2_ as hydrate former. For the model used in this work for CO_2_ the solubility of CO_2_ decreases as the temperature increases,^55^ whereas in ref 58 the solubility of H_2_S increases as *T* increases (see Figure 4d in ref 58). Experimentally the solubility of both guest molecules decreases as T increases, at least under low pressures.^82^ Further studies are needed to clarify whether there are fundamental differences between H_2_S and CO_2_ hydrate nucleation.

In any case, our results challenge the commonly held view that hydrate nucleation universally occurs at interfaces. At high supercooling, bulk (homogeneous) nucleation may in fact dominate. More work is needed to understand the nucleation mechanism at moderate supercooling, where most experimental observations are made.

A promising direction is the direct comparison of nucleation rates from simulations and experiments. This quantity provides a natural bridge between both approaches, allowing validation of simulations, which offer access to molecular-level details that are often inaccessible experimentally. For ice nucleation, the nucleation rate has already been successfully compared between simulations (using the same water model as in the present work) and experiments^75,83^ (even though there is still an open debate over the competition between bulk and surface ice nucleation in small water drops).^84,85^ Unfortunately, for hydrates, very few experimental measurements of nucleation rates are available. In contrast, there are already several simulation studies where hydrate nucleation rates have been calculated. Most of them focus on the bulk, but often using aqueous solutions with unrealistically high hydrate-former concentrations.^29,31,54,55,86^

Our simulations suggest that at sufficiently high supercooling, homogeneous (bulk) nucleation should outpace interfacial nucleation. This regime may offer a valuable opportunity to directly measure homogeneous nucleation rates and compare them with existing simulation predictions, thereby laying a stronger foundation for the molecular-level understanding of hydrate nucleation.

## 5 Summary and Conclusions

We employ molecular dynamics simulations of a realistic water–CO_2_ model to examine the formation of CO_2_ hydrates in supercooled, CO_2_-saturated aqueous solutions at 400 bar. Most simulations are carried out at 255 K, corresponding to a supercooling of 35 K below the hydrate–solution–CO_2_ triple point.^61^ Hydrate seeds are introduced at different positions relative to the interface to monitor their growth. Interestingly, we find that hydrate nucleation proceeds more rapidly in the bulk than near the interface, challenging the conventional view. We also explore spontaneous nucleation pathways (without seeding) at 250 K, a higher supercooling that enables homogeneous hydrate nucleation within our simulation time scale. In this regime, nucleation occurs preferentially in regions with locally enhanced CO_2_ concentration due to thermal fluctuations; these regions arise in the bulk and are not associated with the interface. Our findings highlight the need for careful investigation of hydrate nucleation mechanisms, a process of critical relevance to flow assurance in the oil industry and to natural environmental systems. Further work is required to reconcile these observations with experimental evidence on hydrate nucleation and growth.

## Supplementary Material



## Notes

Additional details on the equilibration protocol used in this study; an extended version of Figure 6 showing hydrate size evolution across all simulations (PDF)

## References

[1] Sloan, E. D. ; Koh, C. Clathrate Hydrates of Natural Gases. 3rd ed.; CRC Press: New York, 2008.

[2] Hirohama S., Shimoyama Y., Wakabayashi A., Tatsuta S., Nishida N. (1996). Conversion
of ch4-hydrate to co2-hydrate in liquid co2. J. Chem. Eng. Jpn.

[3] Park Y., Kim D.-Y., Lee J.-W., Huh D.-G., Park K.-P., Lee J., Lee H. (2006). Sequestering carbon dioxide into complex structures
of naturally occurring gas hydrates. Proc. Natl.
Acad. Sci. U. S. A.

[4] Lee H., Seo Y., Seo Y.-T., Moudrakovski I. L., Ripmeester J. A. (2003). Recovering
methane from solid methane hydrate with carbon dioxide. Angew. Chem., Int. Ed.

[5] Lee S., Lee Y., Lee J., Lee H., Seo Y. (2013). Experimental verification
of methane–carbon dioxide replacement in natural gas hydrates
using a differential scanning calorimeter. Environ.
Sci. Technol.

[6] Sloan E. D. (2021). Hydrocarbon
hydrate flow assurance history as a guide to a conceptual model. Molecules.

[7] Lekvam K., Ruoff P. (1993). A reaction kinetic
mechanism for methane hydrate formation in liquid
water. J. Am. Chem. Soc.

[8] Devarakonda S., Groysman A., Myerson A. S. (1999). THF–water
hydrate crystallization:
an experimental investigation. J. Cryst. Growth.

[9] Takeya S., Hori A., Hondoh T., Uchida T. (2000). Freezing-memory effect
of water on nucleation of CO_2_ hydrate crystals. J. Phys. Chem. B.

[10] Herri J. M., Pic J. S., Gruy F., Cournil M. (1999). Methane hydrate crystal-
lization mechanism from in-situ particle sizing. AIChE J.

[11] Abay H. K., Svartaas T. M. (2011). Multicomponent gas
hydrate nucleation: The effect of
the cooling rate and composition. Energy Fuels.

[12] Jensen L., Thomsen K., von Solms N. (2008). Propane hydrate
nucleation: Experimental
investigation and correlation. Chem. Eng. Sci.

[13] Maeda N. (2018). Nucleation
curves of methane hydrate from constant cooling ramp methods. Fuel.

[14] Maeda N., Shen X. D. (2019). Scaling laws for
nucleation rates of gas hydrate. Fuel.

[15] Liang R., Xu H., Shen Y., Sun S., Xu J., Meng S., Shen Y. R., Tian C. (2019). Nucleation
and dissociation of methane
clathrate embryo at the gas–water interface. Proc. Natl. Acad. Sci. U. S. A.

[16] Maeda N. (2015). Nucleation
curves of model natural gas hydrates on a quasi-free water droplet. AIChE J.

[17] Stoporev A. S., Semenov A. P., Medvedev V. I., Sizikov A. A., Gushchin P. A., Vinokurov V. A., Manakov A. Y. (2018). Visual observation of gas hydrates
nucleation and growth at a water–organic liquid interface. J. Cryst. Growth.

[18] Adamova T. P., Stoporev A. S., Manakov A. Y. (2018). Visual
studies of methane hydrate
formation on the water–oil boundaries. Cryst. Growth Des.

[19] Li C., Metaxas P. J., Barwood M. T., Johns M. L., Aman Z. M., May E. F. (2025). Dependence of gas hydrate formation kinetics on system
size from lag time experiments in a stirred pipe. Energy Fuels.

[20] Jeong K., Metaxas P. J., Helberg A., Johns M. L., Aman Z. M., May E. F. (2022). Gas hydrate nucleation in acoustically levitated water
droplets. Chem. Eng. J.

[21] Sloan, E. D., Jr. ; Koh, C. A. Clathrate hydrates of natural gases. CRC press: 2007.

[22] Kashchiev D., Firoozabadi A. (2002). Nucleation
of gas hydrates. J.
Cryst. Growth.

[23] Báez L. A., Clancy P. (1994). Computer simulation of the crystal
growth and dissolution
of natural gas hydrates. Ann. N.Y. Acad. Sci.

[24] Rodger P. M., Forester T. R., Smith W. (1996). Simulations
of the methane hydrate/methane
gas interface near hydrate forming conditions. Fluid Phase Equilib.

[25] Alavi S., Ripmeester J. A., Klug D. D. (2005). Molecular-dynamics study of structure
II hydrogen clathrates. J. Chem. Phys.

[26] Alavi S., Ripmeester J. A., Klug D. D. (2006). Molecular-dynamics simulations of
binary structure II hydrogen and tetrahydrofurane clathrates. J. Chem. Phys.

[27] Walsh M. R., Koh C. A., Sloan E. D., Sum A. K., Wu D. T. (2009). Microsec-
ond simulations of spontaneous methane hydrate nucleation and growth. Science.

[28] English N. J., Tse J. S. (2009). Mechanisms for thermal
conduction in methane hydrate. Phys. Rev. Lett.

[29] Walsh M. R., Beckham G. T., Koh C. A., Sloan E. D., Wu D. T., Sum A. K. (2011). Methane hydrate
nucleation rates from molecular dynamics
simula- tions: Effects of aqueous methane concentration, interfacial
curvature, and system size. J. Phys. Chem. C.

[30] Sarupria S., Debenedetti P. G. (2012). Homogeneous
nucleation of methane hydrate in microsecond
molecular dynamics simulations. J. Phys. Chem.
Lett.

[31] Knott B. C., Molinero V., Doherty M. F., Peters B. (2012). Homogeneous nucleation
of methane hydrates: Unrealistic under realistic conditions. J. Am. Chem. Soc.

[32] Liang S., Kusalik P. G. (2013). Nucleation of gas hydrates within constant energy systems. J. Phys. Chem. B.

[33] Barnes B.
C., Knott B. C., Beckham G. T., Wu D., Sum A. K. (2014). Molecular
dynamics study of carbondioxide hydrate dissociation. J. Phys. Chem. B.

[34] Yuhara D., Barnes B. C., Suh D., Knott B. C., Beckham G. T., Yasuoka K., Wu D., Sum A. K. (2015). Nucleation rate
analysis of methane hydrate from molecular dynamics simualtions. Faraday Discuss.

[35] Zhang Z., Liu C.-J., Walsh M. R., Guo G.-J. (2016). Effects of ensembles
on methane hydrate nucleation kinetics. Phys.
Chem. Chem. Phys.

[36] Lauricella M., Ciccotti G., English N. J., Peters B., Meloni S. (2017). Mechanisms
and nucleation rate of methane hydrate by dynamical nonequilibrium
molecular dynamics. J. Phys. Chem. C.

[37] Arjun, Berendsen T. A., Bolhuis P. G. (2019). Unbiased atomistic
insight in the competing nucleation
mechanisms of methane hydrates. Proc. Natl.
Acad. Sci. U. S. A.

[38] Arjun A., Bolhuis P. G. (2020). Rate prediction
for homogeneous nucleation of methane
hydrate at moderate supersaturation using transition interface sampling. J. Phys. Chem. B.

[39] Arjun A., Bolhuis P. G. (2021). Homogenous nucleation rate of CO_2_ hydrates
using transition interface sampling. J. Chem.
Phys.

[40] Arjun A., Bolhuis P. G. (2023). Homogeneous nucleation of crystalline methane hydrate
in molecular dynamics transition paths sampled under realistic conditions. J. Chem. Phys.

[41] Zhang Z., Kusalik P. G., Guo G.-J. (2018). Molecular
insight into the growth
of hydrogen and methane binary hydrates. J.
Phys. Chem. C.

[42] Zhang Z., Guo G.-J., Wu N., Kusalik P. G. (2020). Molecular
insights
into guest and composition dependence of mixed hydrate nucleation. J. Phys. Chem. C.

[43] Wang J.-L., Sadus R. J. (2003). Phase Behaviour of Binary Fluid Mixtures:
a Global
Phase Diagram Solely in Terms of Pure Component Properties. Fluid Phase Equilib.

[44] Jiménez-Ángeles F., Firoozabadi A. (2014). Nucleation
of methane hydrates at moderate subcooling
by molecular dynamics simulations. J. Phys.
Chem. C.

[45] Jiménez-Ángeles F., Firoozabadi A. (2018). Hydrophobic hydration and the effect of NaCl salt in
the adsorption of hydrocarbons and surfactants on clathrate hydrates. ACS Cent. Sci.

[46] Zhang Z.-c., Wu N.-y., Liu C.-l., Hao X.-l., Zhang Y.-c., Gao K., Peng B., Zheng C., Tang W., Guo G.-j. (2022). Molecular
simulation studies on natural gas hydrates nucleation and growth:
A review. China Geol.

[47] Tanaka H., Matsumoto M., Yagasaki T. (2023). On the phase behaviors of CH_4_–CO_2_ binary clathrate hydrates: Two-phase
and three-phase coexistences. J. Chem. Phys.

[48] Tanaka H., Matsumoto M., Yagasaki T. (2024). Cage occupancies of CH_4_, CO_2_, and Xe hydrates: Mean field theory and grandcanonical
Monte Carlo simulations. J. Chem. Phys.

[49] Conde M. M., Vega C. (2010). Determining the three-phase coexistence line in methane hydrates
using computer simulations. J. Chem. Phys.

[50] Míguez J. M., Conde M. M., Torré J.-P., Blas F. J., Piñeiro M. M., Vega C. (2015). Molecular dynamics simulation of CO_2_ hydrates: Prediction
of three phase coexistence line. J. Chem. Phys.

[51] Blazquez S., Algaba J., Míguez J. M., Vega C., Blas F. J., Conde M. M. (2024). Three-phase equilibria of hydrates from computer simulation.
I. Finite-size effects in the methane hydrate. J. Chem. Phys.

[52] Algaba J., Blazquez S., Feria E., Míguez J. M., Conde M. M., Blas F. J. (2024). Three-phase equilibria
of hydrates
from computer simulation. II. Finite-size effects in the carbon dioxide
hydrate. J. Chem. Phys.

[53] Waage M. H., Vlugt T. J. H., Kjelstrup S. (2017). Phase diagram
of methane and carbon
dioxide hydrates computed by Monte Carlo simulations. J. Phys. Chem. B.

[54] Grabowska J., Blázquez S., Sanz E., Noya E. G., Zerón I. M., Algaba J., Míguez J. M., Blas F. J., Vega C. (2023). Homogeneous
nucleation rate of methane hydrate formation under experimental conditions
from seeding simulations. J. Chem. Phys.

[55] Zerón I. M., Algaba J., Míguez J. M., Grabowska J., Blazquez S., Sanz E., Vega C., Blas F. J. (2025). Homogeneous
nucleation rate of carbon dioxide hydrate formation under experimental
condition from seeding simulations. J. Chem.
Phys.

[56] Li L., Zhong J., Yan Y., Zhang J., Xu J., Francisco J. S., Zeng X. C. (2020). Unraveling nucleation pathway in
methane clathrate formation. Proc. Natl. Acad.
Sci. U. S. A.

[57] Jacobson L. C., Hujo W., Molinero V. (2010). Amorphous precursors in the nucleation
of clathrate hydrates. J. Am. Chem. Soc.

[58] Zhang Z., Kusalik P. G., Guo G.-J., Li Y., Huang L., Wu N. (2025). Temperature-controlled gas hydrate
nucleation in the heterogeneous
environment. J. Phys. Chem. Lett.

[59] Abascal J. L. F., Sanz E., García
Fernández R., Vega C. (2005). A potential model for the study of
ices and amorphous water: TIP4P/Ice. J. Chem.
Phys.

[60] Potoff J. J., Siepmann J. I. (2001). Vapor-liquid equilibria of mixtures containing alkanes,
carbon dioxide, and nitrogen. AIChE J.

[61] Algaba J., Zerón I. M., Míguez J. M., Grabowska J., Blazquez S., Sanz E., Vega C., Blas F. J. (2023). Solubility
of carbon dioxide in water: Some useful results for hydrate nucleation. J. Chem. Phys.

[62] Blazquez S., Conde M. M., Vega C. (2024). Solubility
of CO_2_ in salty
water: adsorption, interfacial tension and salting out effect. Mol. Phys.

[63] van
der Spoel D., Lindahl E., Hess B., Groenhof G., Mark A. E., Berendsen H. J. (2005). Gromacs: Fast, flexible, and free. J. Comput. Chem.

[64] Hess B., Kutzner C., Van Der Spoel D., Lindahl E. (2008). Gromacs 4: algorithms
for highly efficient, load-balanced, and scalable molecular simulation. J. Chem. Theory Comput.

[65] Nosé S. (1984). A molecular
dynamics method for simulations in the canonical ensemble. Mol. Phys.

[66] Hoover W. G. (1985). Canonical
dynamics: Equilibrium phase-space distributions. Phys. Rev. A.

[67] Parrinello M., Rahman A. (1981). Polymorphic transitions
in single crystals: A new molecular
dynamics method. J. Appl. Phys.

[68] Darden T., York D., Pedersen L. (1993). Particle mesh
ewald: An n·log­(n)
method for ewald sums in large systems. J. Chem.
Phys.

[69] Humphrey W., Dalke A., Schulten K. (1996). VMD –
Visual Molecular Dy-
namics. J. Mol. Graphics.

[70] Becker R., Döring W. (1935). Kinetische
behandlung der keimbildung in übersättigten
dämpfen. Ann. Phys.

[71] Volmer M., Weber A. (1926). Keimbildung in übersättigten
gebilden. Z. Phys. Chem.

[72] Gibbs, J. W. On the equilibrium of heterogeneous substances. Transactions of the Connecticut Academy of Arts and Sciences, 1876, 3, 108–248

[73] Gibbs, J. W. On the equilibrium of heterogeneous substances. Transactions of the Connecticut Academy of Arts and Sciences, 1878, 16, 343–524

[74] Sanz E., Vega C., Espinosa R., Caballero-Bernal R., Abascal J. L. F., Valeriani C. (2013). Homogeneous
Ice Nucleation at Moderate
Supercooling from Molecular Simulation. J. Am.
Chem. Soc.

[75] Niu H., Yang Y. I., Parrinello M. (2019). Temperature
dependence of homogeneous
nucleation in ice. Phys. Rev. Lett.

[76] Hu W., Chen C., Sun J., Zhang N., Zhao J., Liu Y., Ling Z., Li W., Liu W., Song Y. (2022). Three-body
aggregation of guest molecules as a key step in methane hydrate nucleation
and growth. Commun. Chem.

[77] Barnes B. C., Beckham G. T., Wu D. T., Sum A. K. (2014). Two-component
order
parameter for quantifying clathrate hydrate nucleation and growth. J. Chem. Phys.

[78] Vatamanu J., Kusalik P. G. (2010). Observation of two-step
nucleation in methane hydrates. Phys. Chem.
Chem. Phys.

[79] Metaxas P.
J., Lim V. W., Booth C., Zhen J., Stanwix P. L., Johns M. L., Aman Z. M., Haandrikman G., Crosby D., May E. F. (2019). Gas hydrate
formation probability
distributions: Induction times, rates of nucleation and growth. Fuel.

[80] Davies S. R., Hester K. C., Lachance J. W., Koh C. A., Sloan E. D. (2009). Studies
of hydrate nucleation with high pressure differential scanning calorimetry. Chem. Eng. Sci.

[81] Bai D., Chen G., Zhang X., Sum A. K., Wang W. (2015). How properties
of solid surfaces modulate the nucleation of gas hydrate. Sci. Rep.

[82] Sander R. (2015). Compilation
of henry’s law constants (version 4.0) for water as solvent. Atmos. Chem. Phys.

[83] Espinosa J. R., Vega C., Sanz E. (2018). Homogeneous ice nucleation
rate in
water droplets. J. Phys. Chem. C.

[84] Sun G., Tanaka H. (2024). Surface-induced water
crystallisation driven by precursors
formed in negative pressure regions. Nat. Commun.

[85] Haji-Akbari A., Debenedetti P. G. (2017). Computational investigation of surface
freezing in
a molecular model of water. Proc. Natl. Acad.
Sci. U. S. A.

[86] Warrier P., Khan M. N., Srivastava V., Maupin C. M., Koh C. A. (2016). Overview:
Nucleation of clathrate hydrates. J. Chem. Phys.

