# Supplementary material for: Bulk versus Interface Nucleation of CO2 Hydrates from Computer Simulations
Source: J Phys Chem B. 2026 Mar 23;130(13):3717–28. doi: 10.1021/acs.jpcb.5c07607 (PMC13298905; doi:10.1021/acs.jpcb.5c07607)
Supplement: Supplementary file 1 [file jp5c07607_si_001.pdf]

Supporting Information

Bulk versus interface nucleation of CO_2 hydrates from computer simulations.

Joanna Grabowska,[*,†] Samuel Blazquez,[‡] Carlos Vega,[‡] and Eduardo Sanz[*,‡]

†*Department of Physical Chemistry, Faculty of Chemistry, Gdansk University of Technology, ul. Narutowicza 11/12, 80-233 Gdansk, Poland*

‡*Dpto. Química Física I, Fac. Ciencias Químicas, Universidad Complutense de Madrid, 28040 Madrid, Spain*

E-mail: joanna.grabowska@pg.edu.pl; esa01@quim.ucm.es

S1. Equilibration protocols

The interface between the inserted hydrate seed and the surrounding liquid was equlibrated via three different protocols:

- EQ1: all molecules in the inserted seed (all atoms in water and CO_2 molecules) were kept at fixed positions during 1 ns of equilibration
- EQ2: only CO_2 molecules (all of their atoms) in the seed were kept at fixed positions during 3 ns of equilibration
- EQ3: all molecules in the inserted seed (all atoms in water and CO_2 molecules) were kept at fixed positions during 20 ns.

In all cases, the NVT ensemble was used during the equilibration of the interface. In EQ1 and EQ2 equilibration protocols we used different starting radii of the seeds inserted into the systems, ranging from 1.3 to 2.0 nm. In EQ3, since different trajectories evolve differently, we get different radii at the end of equilibration period, depending on the selected trajectory even though all trajectories start from the same radius (1.3 nm).

Fig. S1 shows the number of molecules in the hydrate versus time for different seed placements (ordered in columns) and seed sizes (ordered in rows). The color of the trajectory indicates the equilibration protocol (green corresponds to EQ1, blue to EQ2 and red to EQ3). The number of trajectories corresponding to EQ3 is limited because it requires a long simulation time (20 ns) as compared to the other protocols (1 and 3 ns for EQ1 and EQ2, respectively). As can be seen, there is no indication of correlation between equilibration protocol and tendency to grow or melt of a seed with a given size and location. Accordingly, we compute the probability of growth in the main paper using all trajectories, regardless their equilibration protocol.

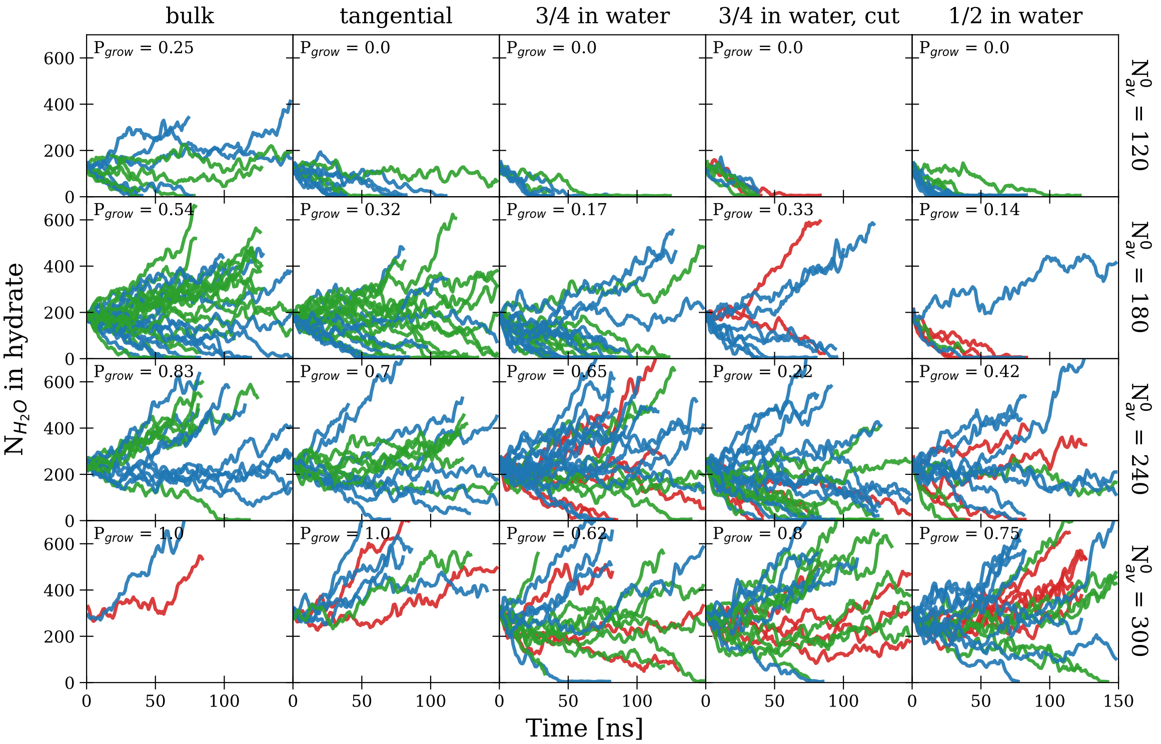

Figure S1: The recolored version of Figure 5 from the main text, where the lines are colored differently based on the equilibration protocol: green lines - EQ1, blue lines - EQ2 and red lines - EQ3.

S2. Extended version of Fig. 6 from the main text

We present here extended version of Fig. 6 from the main text which include all of the initial cluster sizes considered in this work (Fig. S2). For each plot, the probability of growing of the seed of given size and system type is shown in the upper left corner. Presented values were used in Figure 7 in the main text and for the estimation of the critical size of the seed for each system type.

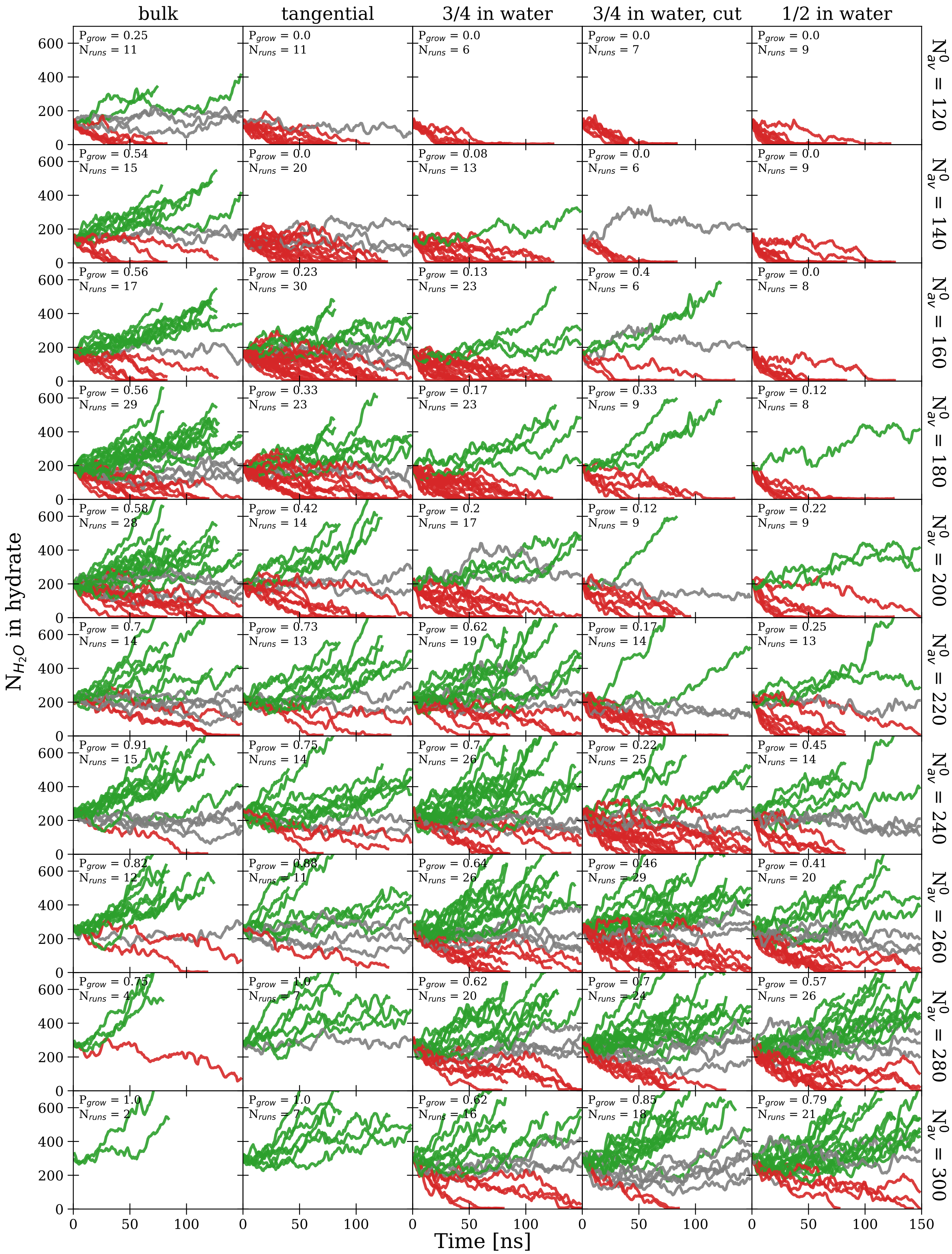

Figure S2: A full version of Fig. 6 from the main text. The coloring scheme is identical to Fig. 6
